# Assessment of the Range of Movement of the Lower Limb in Sport: Advantages of the ROM-SPORT I Battery

**DOI:** 10.3390/ijerph17207606

**Published:** 2020-10-19

**Authors:** Antonio Cejudo, Pilar Sainz de Baranda, Francisco Ayala, Mark De Ste Croix, Fernando Santonja-Medina

**Affiliations:** 1 Department of Physical Activity and Sport, Faculty of Sport Sciences, Regional Campus of International Excellence “Campus Mare Nostrum”, University of Murcia, San Javier, 30720 Murcia, Spain; antonio.cejudo@um.es (A.C.); franciscoayalarodriguez@gmail.com (F.A.); 2 Sports and Musculoskeletal System Research Group (RAQUIS), Regional Campus of International Excellence “Campus Mare Nostrum”, University of Murcia, 30720 Murcia, Spain; mdestecroix@glos.ac.uk (M.D.S.C.); santonja@um.es (F.S.-M.); 3 School of Sport and Exercise, Exercise and Sport Research Centre, University of Gloucestershire, Gloucester GL2 9HW, UK; 4 Department of Surgery, Pediatrics, Obstetrics and Gynecology, Faculty of Medicine, Regional Campus of International Excellence “Campus Mare Nostrum”, University of Murcia, 30100 Murcia, Spain

**Keywords:** flexibility, lumbar support, inclinometer, reliability, injury prevention, performance

## Abstract

Range of movement (ROM) assessment is an important strategy to increase physical-technical performance and minimize the risk of sports-related injuries. Currently, there is no consensus regarding which ROM assessment method is the most appropriate. The main objective of this study was to perform a systematic review of the test batteries available for the assessment of lower limb ROM; additionally, we compare the ROM-SPORT I battery with those previously reported in the literature. The systematic review was conducted following the Preferred Reporting Items for Systematic Review and Meta-Analyses (PRISMA) guidelines. The identification of publications was made by using the databases SciELO, Medline, Scopus, PubMed, and Web of Science. Based on the inclusion criteria, sixteen publications were selected and analyzed. The ROM-SPORT I battery is the most valid of the analyzed methods. This battery evaluates the ROM of eleven lower limb movements. The inclinometer with a telescopic arm and a box is a simpler, more comfortable, and faster procedure than others. The Lumbosant support and use of two examiners are essential to avoid compensatory movements to obtain reliable measurements during ROM assessment. The ROM-SPORT I is a field-based battery of tests that may be used by sports professionals, clinics, and researchers in applied settings to accurately assess and monitor lower extremity ROM.

## 1. Introduction

Flexibility, which is defined as the intrinsic ability of tissues to achieve the maximum range of movement without sports injury [1], is one of the key components of athletic performance together with strength, endurance, speed, and coordination [2,3,4,5]. The range of motion (ROM) in quantitative terms (degrees) represents the indirect measurement of muscle extensibility [1,6,7].

A limited or restricted ROM has been considered an important intrinsic and modifiable risk factors for the most prevalent sports-related injuries, such as groin pain (limited hip adductor [8,9], and internal rotation ROMs [10,11]; hamstring (limited hip flexion ROM [12], and quadriceps (limited knee flexion ROM [10]) muscle strains; patellar (limited hip flexion ROM [13,14]) and Achilles (limited ankle dorsiflexion ROM [15]) tendinopathies; anterior cruciate ligament injury (limited hip rotation [11,15]); as well as lower back pain (limited hip flexion, extension and/or internal rotation ROMs [16,17,18], and femoropatellar pain (limited hip flexion ROM [19]).

A possible explanation for the association between limited ROM and injury risk is attributed to the fact that athletes with limited ROMs have muscle-tendon units that may not be sufficiently prepared to store and release the high amount of elastic energy generated during repeated high-intensity movements that are intrinsic to most sports (e.g., sudden acceleration and deceleration, rapid changes of directions, jumping and landing tasks), and this might predispose such players to high injury risk [12]. Likewise, limited lower extremity joint ROM (e.g., limited hip and knee flexion, and ankle dorsiflexion ROMs) may lead athletes to adopt aberrant movement patterns (e.g., excessive dynamic valgus motion at the knee) during the execution of such high-intensity dynamic tasks (e.g., cutting and landing), which is suggested to increase the risk of soft-tissue (muscle, tendon, and ligament) overloading [20,21,22].

In addition, the ROM can be decreased by high training loads and repeated movements used in technical sports actions during both training and competition throughout the season, which induces physical stress and fatigue on the muscles [23,24]. When these effects are not compensated with adequate recovery measures, the muscle, tendon units may suffer alterations in their mechanical and neuronal properties, including muscle tightness and a ROM reduction [25,26,27]. Low ROM values are related to higher sports injury, but a high ROM value does not ensure injury prevention, because injury risk is a complex and multifactorial issue. Although it is generally assumed that those competing at the highest sport levels have higher ROM values [5,28,29], a high incidence of severe sports injuries has been shown in the highest level of competition for rowing, ice-hockey, football, and rugby [30,31,32,33]. Furthermore, it has been demonstrated that high ROM values are required to adequately perform the highly demanding technical actions of gymnastics, taekwondo, diving, and figure skating [17,34,35]; in this sense, a limited ROM decreases physical performance in these sports [3,36,37,38,39]. Therefore, it is clearly necessary to assess the athlete’s ROM (especially in the major joints of the lower extremity or poly-articular muscles, due to their high rate of injuries) not only to prevent injuries, but also in certain sports, as a quantifiable training component that may be fundamental to achieving a high level of sports-related performance.

Certain articles in the scientific literature that assess athletes´ flexibility show major differences in results depending on the sport [4,34,35,40,41]. Thus, we can observe that flexibility is specific for each joint, muscle action, or movement and that for the same sport, different joints require differing degrees of flexibility [17,42]. Flexibility also differs depending on the specific position of each player in a team [43,44], between the dominant and non-dominant limb [45,46,47], and at varying competitive levels [5,28,29]. For example, the study of Gannon et al. [48] indicated that international athletes (dancers and gymnasts) present higher flexibility values (shoulder flexion and extension ROM, hip flexion, extension and abduction ROM with full knee extension, trunk ROM and ankle ROM) than national, beginners or active athletes. Moreno et al. [35] on estimating hamstring muscle flexibility using the sit and reach (SRT) test in 32 different sports, concluded that elite athletes present higher flexibility values than the general population. Battista et al. [28] concluded that university rowers present better hamstring flexibility (SRT) than amateurs, and that experienced rowers have higher flexibility [28]. De la Fuente and Gómez-Landero [5] examined the differences between Taekwondo cadet-athletes competing at different competitive levels and found that hip ROM was significantly different between medalists and non-medalists in both the passive flexion test of both legs and the abduction test in active and passive positions.

There are many published assessment tests to measure the ROM of the major joints in the lower extremities (i.e., hip, knee, and ankle) [12,49,50,51,52,53,54,55,56]. Subsequently, there are several different methodologies used to assess ROM, for example, passively (e.g., straight leg raise test [hip flexion ROM] or actively (i.e., walking step test [ankle dorsiflexion ROM], and/or using single (Thomas test [hip extension ROM]) or multi (deep back squat [hip flexion ROM] joints. Furthermore, numerous instruments have been suggested to aid measurement of ROM directly (Leighton flexometer, inclinometer or goniometer) or indirectly (measuring tape, video camera) in degrees. However, and despite the large number of published ROM tests, there is currently no consensus as to what exploratory tests are the most appropriate to assess the ROM of the major lower extremity joints [6,57]. The selection of a reference diagnostic, based on the suggestions of Hopkins [58,59], should be based firstly on the criteria of high validity and reliability, and then to value simplicity and universality of the procedure. The identification of the criterion-referenced assessment tests and the promotion of their use in differing contexts would allow practitioners to unify ROM assessment and monitoring.

The ROM-SPORT I battery is a ROM assessment method that has been used to assess the main movements of the lower limb (11 ROM tests for hip [*n* = 7], knee [*n* = 1], and ankle [*n* = 2]) in athletes and general population [7,24,60,61]. Currently, it seems that this battery may be the most appropriate in terms of validity, reliability, simplicity of the procedure, and low requirements of human and material resources. Therefore, the main objective of this study was to perform a systematic review of the batteries available for the assessment of the lower limb ROM; and secondly to compare the ROM-SPORT I battery with those previously reported in the literature. This review may be of importance for researchers working in sports performance, risk of sports injuries, clinical evaluation, and others. In addition, complete information about the existing ROM batteries would also be useful for sports professionals, clinics, and athletes.

## 2. Methods

### Systematic Review

**Figure 1:**
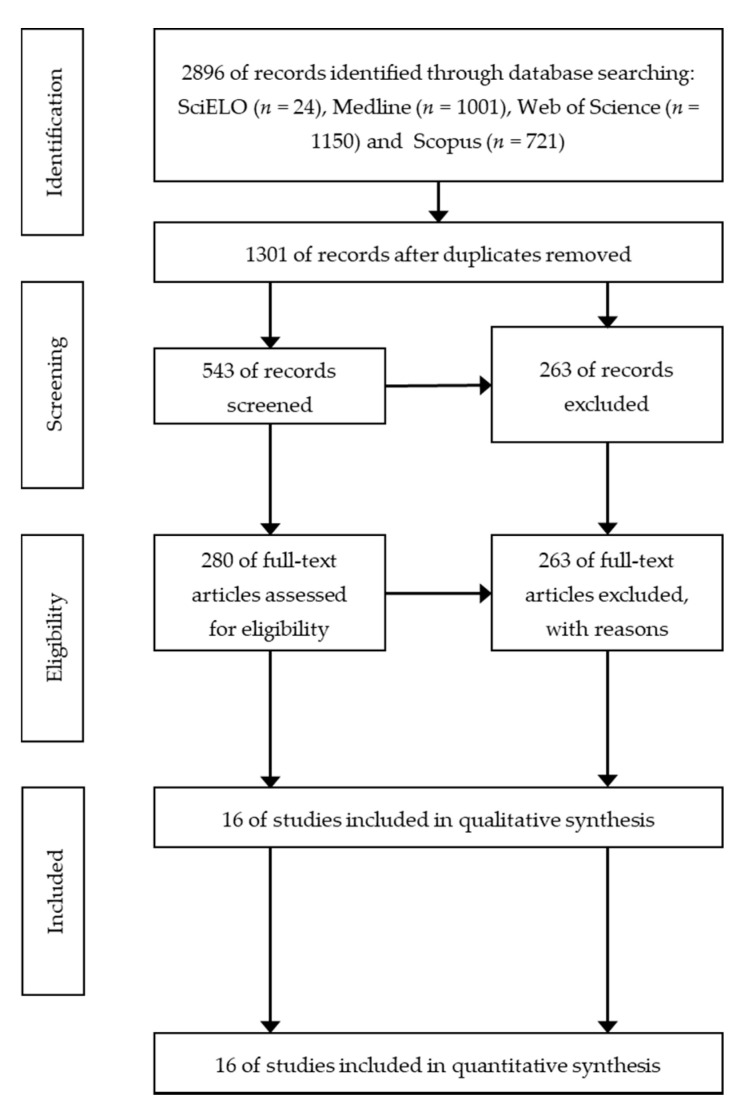
Flow diagram of the systematic review process.

The systematic review was conducted following the Preferred Reporting Items for Systematic Reviews (PRISMA) guidelines [62]. According to the PRISMA Statement guidelines to conduct a systematic review [62], which include the following four steps: Identification, screening, eligibility, and inclusion (Figure 1).

The systematic computerized search was conducted up to 5th August 2020. The identification of publications was made by using the databases Google Scholar, SciELO, Medline, Scopus, PubMed, and Web of Science. The search strategy for identification used the Boolean constructs and combinations of the relevant keywords “flexibility”, “range of motion”, “Range of movement”, and “ROM”. The descriptors “flexibility”, “range of motion”, and “Range of movement” were used with the search operators “OR” and “AND”.

The purpose of the first screening of publications was to select those written in English and to discard publications in the form of literature reviews, abstracts, editorial commentaries, and letters to the editor. Then, the eligibility process for publications was performed according to the following inclusion criteria: (1) Being published before August 2020, (2) research studies assessing the ROM of lower limbs, (3) studies including a battery with at least four ROM tests, (4) articles describing the tests including details, such as starting and final position, type of movement, compensation control, repetitions or trials, human and material resources, (5) using a sample of at least 15 participants, and (6) reporting the reliability.

Two independent reviewers (A.C. and P.S.B.) selected the publications that met the searching, screening, and inclusion criteria. Disagreements were resolved by consulting a third reviewer (F.S.M.).

The variables that were obtained from those publications chosen in this systematic review (data extraction) were classified into twelve categories: (1) General descriptors (authors and publication year), (2) estimated time for testing, (3) warm-up regimen before testing, (4) participant´s starting position, (5) movement testing, (6), measurement procedure (instruments and human resources), (7) types of range of motion evaluated, (8), criteria for end-of test, (9) control of compensatory movements, (10), number of assessment sessions and repetitions for each ROM test, (11) validity and (12) reliability. These categories are considered the most important features are describing the ROM measurement methods [6,57,63]. The ROM-SPORT I battery is a sport-specific ROM assessment tool [40,49,63]. In order to investigate the possible advantages of the ROM-SPORT I battery, this battery was compared with the rest of the ROM assessment batteries published according to these 12 categories.

## 3. Results

A total of 2896 publications were initially identified using Google Scholar, SciELO, Medline, Scopus, PubMed, and Web of Science. At the end of the screening and eligibility processes based on the inclusion criteria, sixteen publications were selected and analyzed (Figure 1). These sixteen publications describe ROM assessment methods consisting of batteries or groups of ROM tests, each ROM test corresponding to the assessment of a specific joint movement; the extensibility of a muscle and other joint tissues are measured in each joint movement. Table 1 shows the information regarding the 12 descriptive categories of the ROM measurement methods described in the 16 studies selected in the systematic review.

### 3.1. Estimated Time for Testing

Data describing this parameter is very scarce in the literature, and in general, this information is rarely provided. Only Bozic et al. [54] and Cejudo et al. [42,45] indicate the estimated testing time, which was 25 min and 1 min for each ROM test, respectively. The testing duration of the ROM-SPORT I battery (11 ROM tests and both sides of the body) varies from approximately 8–11 min [40,42,45].

### 3.2. Warm-Up before Testing

Six studies reported using a warm-up before measuring the ROM [11,45,54,64,65,67]. No warm-up exercises were undertaken in the studies of Ekstrand et al. [74] and Steinberg at al. [69]. The other selected publications did not provide information relating to a warm-up [12,50,66,70,71,73].

### 3.3. Participant’s Starting Position

Athletes are placed in different starting positions depending on the study. The starting positions described are standing, supine, prone, lateral, and sitting. Testing in a supine position was the most commonly used starting position to assess the ROM, followed by the prone position.

### 3.4. Movement Testing

The assessment batteries selected in this systematic review include between four [12] and eleven [42,45] ROM tests. The ROM tests selected in each study depended on the objective of the study. For hip ROM, the movements tested were: (1) Hip extension with neutral knee, relax knee flexion or 80° knee flexion (supine, sagittal plane) for iliopsoas; (2) hip adduction with 90° hip flexion (supine, transversal plane) for the hip abductors muscles; (3) hip flexion with knee extension or knee extension with 90° hip flexion “Hamstring 90/90” (supine, sagittal plane) for hamstrings; (4) hip flexion with relax flexion knee (supine, sagittal plane) for gluteus maximus; (5) hip abduction with neutral knee (supine, frontal plane) for adductors; (6) hip abduction with 90° knee flexion (supine, transversal plane) for monoarticular adductors; (7) hip internal rotation with neutral hip and 90° knee flexion (prono, transversal plane) or 90° hip and knee flexion (supine, transversal plane) for external rotator muscles; and (8) hip external rotation with neutral hip and 90° knee flexion (prono, transversal plane) or 90° hip and 90° knee flexion (supine, transversal plane) for internal rotator muscles ROM tests. For the knee ROM assessment, the movements tested were: (1) Knee flexion with neutral hip (supine, sagittal plane) for quadriceps; and (2) knee extension (supine, sagittal plane) for hamstrings. The ankle joints ROM were evaluated by testing (1) ankle dorsiflexion with 90° knee flexion or maximum knee flexion (standing, sagittal plane) for soleus, (2) ankle dorsiflexion with neutral knee (standing, sagittal plane) for gastrocnemius, and (3) ankle plantar flexion (supine, sagittal plane) for ankle flexor muscles.

### 3.5. Measurement Procedure (Instruments, Material and Human Resources)

The most commonly used measuring instrument for ROM is the two-armed standard goniometer (GM). In addition, other measurement instruments include certain field based methods—Kinanthropometry, ruler and protractor [54,73], extendable GM [73], Leighton flexometer [74], Orthoranger [66], electronic inclinometer [70], inclinometer with a telescopic rod [45,63], electromagnetic tracking system [56], and video capture digital and software for 2- or 3-dimensional image-based analysis [50,53,54].

All selected studies reported that ROM assessment procedures were performed by two experienced examiners, except for the studies of Pua et al. [70] and Tainaka et al. [71] with only one examiner employed. Routinely, one examiner performs the movement, and the second examiner measures the angle with GM. However, Cejudo et al. [45,63] reported that the main examiner executes the movement and takes the ROM measures, while the assistant examiner maintains the initial position of the subject (zero position) and controls compensatory movements, which is considered a major contribution of their protocol.

Most authors used anatomical landmarks to determine the sides of the ROM angle or to measure the angle from an initial position. When measuring with digital capture, the examiner places reflective skin markers on certain bone points [53,54]. Other examiners simplify the procedure by placing the measuring instrument in the longitudinal axis of the mobilized segment through the imaginary bisector line [45,56,63], instead of using landmarks.

### 3.6. Types of ROM Evaluated

The maximal passive and active ROM are the types of movement used in the studies. Specifically, maximum passive movement is predominantly used in the observed studies (13 out of 16 publications).

### 3.7. Criteria for End-Of Test

Five criteria of end-of ROM test were established by the authors: (1) Feeling of stretching or tolerable stretch, no pain [45,50,63,69], (2) point of resistance, firm or stiff end sensation [40,63,70,71], (3) maximum ROM [11,12,45,54,55,63,64,67], (4) the emergence of compensatory movements [11,12,40,63,65,67], or (5) standardized force application [53,56]. Usually, the authors consider two or three of these end-of ROM test criteria in their studies. Most of these criteria are subjective because they are based on qualitative observations, except the criterion of force application; in this case, the quantification of the applied force determines the test end [53,56].

### 3.8. Control of Compensatory Movements

Only six studies outline information related to the control of compensatory movements during ROM measurements, such as a compensatory trunk, pelvis (rotation, lateral tilting, anterior and posterior pelvis tilt), opposite hip (flexion, rotation, and abduction), knee (flexion and extension) and ankle and foot (pronation, supination, and heel on the ground) movements [45,63,64,65,67,70,74]. Different methods were used to avoid these compensatory movements, including velcro bands or straps [67,70,74], the explored athlete himself [12,45,63,64,74], Bledsoe knee brace [67], and the lumbar support tool, “Lumbosant” (Imucot Traumatología SL, Murcia, Spain), together with an assistant examiner [45,63].

### 3.9. Number of Assessment Sessions and Repetitions

Selected studies reported two or three assessment sessions. It is understood that other studies used a single assessment session [12,50].

These studies described one, two or three repetitions or trials of each ROM test. Studies that do not provide this information are understood to carry out a single repetition [12,53,67,69].

### 3.10. Validity

The validity criterion is not reported by most of the selected studies. Witvrouw et al. [12] and Cejudo et al. [40] report criterion validity (gold standard) of their ROM tests based on previous studies reported by Gogia et al. [68] and Enwemeka et al. [75]. In addition, Cejudo et al. [45,63] report content validity for the battery´s ROM tests taken as reference values from the anatomical knowledge and extensive clinical, and sports experience of two American medical organizations (American Medical Association [6] and American Academy of Orthopaedic Association [76]). The studies of Bozic et al. [54] and Nussbaumer et al. [56] determined the concurrent validity between their digital motion measurement method (kinematic analysis) and field methods.

### 3.11. Reliability

All publications report reliability of their ROM measuring batteries. However, different populations studied, the same examiner or several examiners, research designs, and statistical tests have been used to calculate the reliability values of measurement.

Authors displayed coefficient of variation (CV) values ranging from 1.1 to 2.6% [64,74], Bozic et al. [54] from 2.1% to 6.7%, Nussbaumer et al. [56] from 2.6% to 10.2%, Fourchet et al. [53] from 2.6% to 12.4% and Reid et al. [65] reports an average of 4,3%. In sense, Grazette et al. [73] show CV values between 8.3° to 65.3°.

Grazette et al. [73] reported intraclass correlation coefficients (ICC) values between 0.47 to 0.95, Fourchet et al. between 0.51 to 0.92 [53], Bozic et al. between 0.57 to 0.94 [54], Shah et al. between 0.62 to 0.90 [11], Clapper et al. between 0.72 to 0.95 [66], Nussbaumer et al. between 0.82 to 0.95 [56], Cejudo et al. between 0.89 to 0.96 [63,72], Wang et al. [67] between 0.90 to 0.97, and Bradley and Portas [50] between 0.91 to 0.95.

Pua et al. [70] reported minimum detectable change values (MDC) at a 90% confidence interval between 7.1° to 11°, and Cejudo et al. [52,76] reported MDC at a 95% confidence interval between 3.7° and 6.9°. Lastly, Steinberg et al. [69] reported Pearson r values between 0.89 to 0.96, and Tainaka et al. [71] reported average values of 0.85.

## 4. Discussion

A battery of ROM evaluation tests should be characterized by: (1) Inclusion of measurement of the extensibility of the major joints of the lower extremities (at least 11 ROM tests), (2) a simple exploratory procedure to administer, (3) allows examiners to directly assess (in degrees) the ROM in a very short period of time, (4) austerity in human and material resources that especially aim to avoid compensatory movements, (5) valid ROM test and (6) reliable ROM test. Examiners and researchers should consider the strengths and limitations of each method (Table 1) when measuring ROM in athletes and the general population. This section aims to discuss and analyze the different batteries available for the evaluation of ROM as described in the selected publications according to the optimal characteristics of an assessment procedure.

The estimated time for testing ROM using an inclinometer with a telescopic rod (method of ROM-SPORT I battery) is 1 min per ROM test [45,63]. The ROM-SPORT I battery presents a much faster procedure than the procedures proposed by the other authors. The marking of anatomical landmark, the complexity of using the measuring instrument (GM, electronic computerized goniometer, Leighton flexometer, capture digital, electromagnetic tracking system), and the additional use of hand-held dynamometers leads to a considerable increase in the estimated time for testing—more than 3 min per ROM test [54]. This time is still higher if the ROM is subsequently measured using a digital image or video capture or an electromagnetic tracking system. After obtaining a digital image or video, examiners still need to measure the angle with digital motion analysis software [53,54]; also, a lot of time is spent on locating the body area and attaching the sensors of electromagnetic tracking system with double-sided tape, flexible medical adhesive tape, and a velcro band, all increasing the time needed for the assessment. In addition, the increased complexity of the procedure is associated with an increase in the variability and potential error of the measure [58,77].

The warm-up mainly has three aims: (1) To minimize the risk of muscle injury because all the tests required a large muscle tension stimulus [78,79], (2) to reduce the effects of muscle lengthening on repeated trials during data collection [80,81], and (3) to reduce the variability and standard error of measurements by minimizing the effect of different muscle temperature on muscle flexibility [81] that could be, for example, caused by the different means transport (walking, bike, car…) employed by the athletes to reach de assessment session. However, there are certain circumstances where the examiners do not have sufficient time to warm-up, such as the limited time provided by coaches or the time required for other tests different from flexibility.

Trying to reduce the participant positioning during tests is essential to reduce the time it takes to complete the test battery. In addition, appropriate starting positions and movements (movements contrary to the muscle actions) ensures specific muscle extensibility and ROM measurement [63,82]. Starting or ‘zero position’ [6] that is used in the studies of Nussbaumer et al. [56] and Cejudo et al. [45,63] facilitates the measurement of the ROM only once (at the final test position).

The ROM tests selected in each ROM assessment battery depend on the objective of the study. The main movements of the lower limb are hip extension, hip adduction with the hip flexed 90°, hip flexion with the knee flexed and extended, hip abduction with the hip neutral and hip flexed 90°, hip external and internal rotation, knee flexion, ankle dorsiflexion with the knee flexed and extended ROMs. These movements have usually been selected in the scientific literature because limited ROM, induced by muscle tightness, increases the sports injury risk [24,83] and decreases athletic physical-technical performance [3,36,38]. In addition, the measurement results of each of these ROMs in ascending order determines the lower, limb flexibility profile in the sport and is based on the specificity of this component of physical fitness to a given sport [7,17,42,45,49,83].

The ROM assessment using an inclinometer with a telescopic rod (method of the ROM-SPORT I battery) appears to be a simpler and faster method than using other instruments (Table 1). It has the advantage of not requiring the marking of bony landmarks, since the maximum ROM values can be determined as the angle formed by the longitudinal axis of the leg (lateral or anterior bisector of the leg) within the vertical or horizontal planes. In this sense, the initial and final positions can be identified with systematic and repetitive precision [6,50,63,72,84]. Also, using an inclinometer with a telescopic arm turns the instrument into a one-arm goniometer, with the advantage of having a gravity level that allows for the better precision of measurements, and subsequently, increasing measuring speed [6,63,72]. In addition, this instrument does not have the disadvantage of, for example, the goniometer, which requires the precise positioning of its arms, while moving the goniometer at the same time as the limb [6,63]. Finally, and unlike other more sophisticated tools, the cost of an inclinometer is relatively low (ranging from 110 to 150€).

Two experienced examiners are required to measure hip and knee ROMs. In the measurement with GM participate, two examiners, the main examiner perform the ROM tests movements, while the assistant examiner place the two, armed GM on the two body segments. However, generally, there is no control over the compensatory movements in this method, which may result in an imprecise measure (high standard error of measurement). This is a limitation of the ROM assessment with GM, since it is essential to avoiding compensatory movements, which may result in imprecise measures (high standard error of measurement) [84,85,86]. A recent study by Santonja et al. [84] observed 13.9° less hip flexion with knee extension or “Straight Leg Raising” test when the compensatory movements were not controlled for by the lumbar support tool, “Lumbosant” (Imucot Traumatología SL, Murcia, Spain), together with an assistant examiner. For examiners to take on more competences, Cejudo et al. [63,72,87] recommend using an inclinometer with a telescopic rod, which behaves like a single one-armed GM. This measuring instrument allows the main examiner to perform the movement and measurement of the ROM with the inclinometer, while the assistant examiner avoids the compensatory movements.

The use of anatomical landmarks and the placement of reflective skin markers or Orthoranger pendulum considerably increases the measurement time of any ROM assessment battery. To reduce the time of the measurement procedure, Cejudo et al. [45,63] and Nussbaumer et al. [56] used a movable armed GM or telescopic rod of an inclinometer with a longitudinal axis of the mobilized segment, following its imaginary bisector line. Compared to the GM, the inclinometer with a telescopic rod allows the examiner to simultaneously perform compensatory movement control, movement testing, and ROM measurement [45,63].

The passive maximal measurement is the most commonly used maneuver in the selected studies [11,12,42,53,54,56,63,64,65,67,69,70,73,74]. The use of passive movements is based on the following two reasons: First, in several of the active tests, the peak ROM depends on the participant’s muscle strength (mainly psoas, hamstring, quadriceps) and the ability to simultaneously contract the agonist muscles and relax the antagonist muscles that are to be measured [88]. This makes the application of the active tests very limited in individuals with lower absolute strength of specific muscles, such as children and adolescents [89]. Furthermore, it does not compare the ROM values between sex and sport disciplines, due to the different strength profile of their participants [4]. Second, the active tests are strongly influenced by the participant’s motivation to actively move the joint until achieving the peak ROM, which can be considered a source of error of the measurement (intra-individual variability) [6]. However, it should be noted that the ROM assessment tests of the ankle joint are active, due to the fact that the passive measurements are technically more difficult to carry out, which has shown to negatively influence the precision of the measurement obtained from them [90].

Furthermore, all the selected passive tests specifically measure a single joint movement. The tests that imply using more than one joint (i.e., sit and reach, Functional Movement Screen [FMS^®^]) might not accurately assess ROM, as they may be biased by other factors, such as anthropometry (length of the limbs), and inter-muscular coordination (dynamic stability) which could limit the validity of the results.

The ROM is the measurement of movement around a specific joint in the body. The aim of the ROM measurement is to indirectly quantify muscle extensibility [1,6,63]. Usually, a maximum passive movement is performed by the main examiner [12,45,54,63,66,69,74]. However, the test can be terminated earlier if an examiner felt or appreciated some compensatory movement that may increase the ROM [45,63].

Lastly, the aim of ROM measurement is to quantify muscle extensibility. For that reason, the maximum movements opposed to the actions of the muscle must be performed during the ROM test procedure for the subject or athlete to feel the muscle stretching as the final criterion of the test [45,50,63,69] without reaching the pain point, which can request myotactic reflex.

The control of the applied force during the movement of the ROM test contributes to the standardization of the protocol and adds a new criterion for the end of the ROM test [53,56]. However, the inclusion of this criterion considerably increases the complexity of the procedure and increases the evaluating time and the error of the measure.

Compensatory movements in the trunk, pelvis, opposite lower limb, ankle, and foot are produced during the measurement of lower limb ROM [63,64,65,67,70,74]. The studies of Cejudo et al. [45,63,72] precisely detail the possible compensatory movement pattern in each ROM test of the ROM-SPORT I battery; these authors report that the lumbar support tool, “Lumbosant” (Imucot Traumatología SL, Murcia, Spain), and two examiners can help in minimizing compensatory movements of the hip and knee during ROM tests. For ankle ROM tests, the athlete and the main examiner control the compensatory movements [87]. This method is better in helping to limit compensatory movements of the trunk, pelvis, and lower limb than the use of velcro bands or straps [63,72]. The lumbar support tool, “Lumbosant” (Imucot Traumatología SL, Murcia, Spain), is a reference for the assistant examiner to keep the pelvis in a zero/neutral position [84]. The task of the assistant examiner is to provide the proper stability based on the initial position, by fixing a certain segment of the pelvis throughout the assessment maneuver, to avoid or minimize any compensatory movements, which could increase and bias the outcome. However, using two examiners to carry out the tests appears to limit the practical application of these measurement methods in the sport and clinic context. As these measurement methods are simple to perform, the role of the assistant examiner could be undertaken by any postgraduate student or athletic trainer who performs one or two 10 min training sessions (statement based on the authors’ extensive experience). The Bledsoe knee brace of Wang et al. [67] is an effective device for fixing the knee; however, its use requires extra time.

Several assessment sessions required depends directly on the type of study. Generally, scientific studies perform only one evaluation session; specifically, studies to determine the lower-limb flexibility profile [7,42,45,49,91,92] or to associate/predict athletic physical-technical performance [3,37,39,93] and risk of injury [11,12,24,83,94]. The validity and absolute reliability of scientific studies involved 2–4 assessment sessions to determine the relative or absolute reliability of an assessment battery test [58]. Moreover, studies conducted to determine the chronic effects of a flexibility program on ROM include several assessment sessions [95,96].

A single repetition reported per ROM test by different authors [12,53,67,69] does not provide the examiner with the precision or variability of measurements beyond error [58,97].

The most recommended by authors is to perform at least two repetitions o trials per ROM test [45,63,70]. For that reason, the proposal of Cejudo et al. [45,63] performs two maximal trials of each test and limb (dominant and non-dominant) in a randomized order. The mean score for each test would be considered as the final (true) ROM value. In the cases where variation is higher than 5% in the ROM values between the two trials of any test, an extra trial would be performed [45,63]. The two most closely related trials would be used to calculate the true ROM value, as long as the difference with the new trial is <5%. If this is not the case, then the examiner would be required to revise the procedure for any possible error or review the circumstances that may explain the variability.

The content validity is determined by judging if an instrument or procedure accurately measures and represents the variable of interest. In this sense, all the selected assessment tests (Table 1), including ROM-SPORT I battery tests, have been considered appropriate by the American Medical Organizations [6,76] and included in the accredited manuals of Sports Medicine and Science [6,57,82], based on anatomical knowledge and extensive clinical experience.

Studies based on a radiography method considered the standard criterion (gold standard) for measuring flexibility, report a high concordance (criterion validity or both procedures can be interchangeable) with the ROM measurement method using a goniometer or inclinometer [84,98]. Some studies have examined the criterion-related validity (mainly through correlation coefficients) of some knee and hip ROM measures recorded using different field-based tools (i.e., mainly inclinometers and goniometers) and radiography [68,75]. These ROM measures obtained through using goniometers and inclinometers have reported correlations with their respective radiography criterion measures higher than 0.80, which suggests that their use may be interchangeable [68,75,84]. Finally, studies are needed to determine the criterion-related validity of the digital motion measurement method (kinematic analysis) proposed by Bozic et al. [54] and Nussbaumer et al. [56] in accordance with the radiographic method.

All the tests represented in Table 1 displayed moderate to excellent reliability values (ICC ranging from 0.72 to 0.99) except for hip extension with knee flexion and knee extension with 90° hip flexion tests [53]. This result may possibly be due to the complex procedure of the test and evaluation methods (anatomical landmarks, hand-held dynamometer, video capture digital, and digital motion analysis software).

According to absolute reliability values, clinicians and sport practitioners can be 95% confident that an observer’s change between two measures larger than 1.3–6.9° for the ROM values obtained from the ROM-SPORT I would likely indicate a real change (determined through the statistical minimal detectable change with a 95% confidence interval.

## 5. Practical Guidelines

For the practical application of the ROM-SPORT I battery test, users should consider the following aspects to obtain accurate and valid measurements: (1) The majority of the tests of the ROM-SPORT I battery (9 of the 11) involve two examiners to avoid any possible compensatory movements; it reduces measurement error and may give more accurate ROM values, avoiding false diagnostic of limited ROM; (2) The inclinometer (the instrument used in the ROM-SPORT I procedure) is easy and simple to use, as it does not require the estimation of the joint’s axis nor the initial position. Also, this tool reduces measurement errors since the examiner can systematically and repeatedly locate the same position to place the inclinometer by just following the parallel imaginary bisector of the segment assessed; (3) It is recommended to use a telescopic arm to facilitate the inclinometer’s placement, which may improve the precision and reproducibility of the measurement and reduce the duration of the ROM-SPORT I battery; (4) The lower-back protection support “Lumbosant” (Imucot Traumatología SL, Murcia, Spain), used in the ROM-SPORT I battery, helps to standardize the lumbar curvature (20°) during the assessments, avoiding higher ROM values, due to anterior or posterior pelvis tilt compensatory movements.

Although this review has been focused mainly on a sports context, the ROM-SPORT I battery can also be applied in research (clinical studies, sports performance sport risk injuries, and others) and clinical fields. This battery has the following practical applications:

− To accurately quantify the ROM measures of the major lower extremity joints (hip, knee, and ankle);
− To identify athletes with limited or restricted joint ROM values. This knowledge may help in the decision-making process regarding the identification of athletes at high risk of sustaining an injury (mainly soft tissue injury);
− To detect those athletes (e.g., rhythmic gymnasts, figure skaters, and diving) that should improve their ROM values to successfully perform the technical actions that are awarded the highest points by a judge;
− To monitor the efficacy of intervention programs (e.g., stretching exercises and foam rolling) designed to maintain or improve lower extremity joints ROM;
− Furthermore, in rehabilitation processes, the ROM-SPORT I battery may be used to determine if the ROM of the injured joint has been fully restored, which may help to achieve a safe return to play (athletes) or activities of daily life (general population).

## 6. Conclusions

Although different batteries have been used to assess ROM previously, they all have some limitations. The ROM-SPORT I battery seems to be the most complete procedure that meets the requirements of a battery of tests for the ROM assessment of the lower limbs. The novelty and new contributions of the ROM-SPORT I battery over other procedures described previously by other authors are: (1) The rapidity of the ROM-SPORT I battery. This procedure evaluates 11 tests in one athlete, including both lower limbs in 8–11 min; (2) the importance of the assistant examiner, together with using the lumbar support tool, “Lumbosant” (Imucot Traumatología SL, Murcia, Spain), to reduce compensatory movements; (3) the simplicity of the procedure. The inclinometer with an extensible rod is a simple and cheap tool that minimizes the measured variability and the error of the examiner.; (4) the validity of the ROM-SPORT I battery is based on criterion validity (radiographic) and content validity; and (5) all the ROM tests of the selected publications reported moderate to excellent reliability values; it is excellent for tests of ROM-SPORT I battery.

## Figures and Tables

**Table 1:** A summarized of all the variables studied in the 16 selected publications of the batteries for the assessment of the lower limb range of movement.

Reference	Estimate Time for Testing	Warm-up before Testing	Participant´s Starting Position	Movement Testing	Measurement Procedure (Instruments, Material and Human Resources)	Types of Range of Movement (ROM) Evaluated	Criteria for End-of Test	Control of Compensatory Movements	Number of Assessment Sessions and Repetitions	Validity	Reliability
Ekstrand et al. [55]	No detail	No	Supine	Hip extension	2-examiners Velcro bands Anatomical landmark Standard GM Leighton flexometer	Passive	Maximum ROM	APT	2 testing sessions 1 rep	No detail	CV = 1.2%
			Supine	Hip flexion, knee extension				PPT Contralateral hip flexion			CV = 1.4%
			Supine	Hip abduction, knee flexion				No detail			CV = 2.5%
			Supine	Knee flexion				APT			CV = 1.1%
			Standing	Ankle dorsiflexion, neutral knee				Knee flexion, heel on the ground			CV = 2.5%
			Standing	Ankle dorsiflexion, maximum knee flexion				Heel on the ground			CV = 2.6%
Moller et al. [64]	No detail	Yes	Supine	Hip extension	2-examiners Velcro bands Anatomical landmark Standard GM	Passive	Maximum ROM	APT	2 testing sessions 1 rep	No detail	CV = 1.2%
			Supine	Hip flexion, knee extension				PPT, contralateral hip flexion			CV = 1.4%
			Supine	Hip abduction, knee flexion				No detail			CV = 2.5%
			Supine	Knee flexion				APT			CV = 1.1%
			Standing	Ankle dorsiflexion, neutral knee				Knee flexion, heel on the ground			CV = 2.5%
			Standing	Ankle dorsiflexion, maximum knee flexion				Heel on the ground			CV = 2.6%
Reid et al. [65]	No detail	Yes	Supine	Hip extension, knee relax	2-examiners Velcro bands Anatomical landmark Standard GM	Passive	Compensation movements	APT	3 testing sessions, alternate days 3 reps	No detail	CV = 4.3%
			Supine	Hip flexion, knee flexion			No detail	No detail			
			Supine	Hip abduction, neutral knee			No detail	No detail			
			Lateral	Hip adduction (Ober test)			No detail	No detail			
			Sitting	Hip internal rotation			No detail	No detail			
			Sitting	Hip external rotation			No detail	No detail			
Clapper et al. [66]	No detail	No detail	Supine	Hip flexion	2-examiners Anatomical landmark Standard GM Orthoranger pendulum oriented perpendicular to the long axis of the moving segment	Active	No detail	No detail	3 testing sessions (3-week apart) 3 reps	No detail	ICC GM = 0.95 ICC ORR = 0.89
			Prone	Hip extension, neutral knee							ICC GM = 0.83 ICC ORR = 0.72
			Standing	Hip abduction							ICC GM = 0.86 ICC ORR = 0.79
			Standing	Hip adduction							ICC GM = 0.80 ICC ORR = 0.77
			Supine	Hip internal rotation							ICC GM = 0.92 ICC ORR = 0.86
			Supine	Hip external rotation							ICC GM = 0.80 ICC ORR = 0.86
			Supine	Knee flexion							ICC GM = 0.95 ICC ORR = 0.91
			Supine	Knee extension							ICC GM = 0.85 ICC ORR = 0.80
			Supine	Ankle dorsiflexion							ICC GM = 0.92 ICC ORR = 0.80
			Supine	Ankle plantar flexion							ICC GM = 0.96 ICC ORR = 0.93
Wang et al. [67]	No detail	Yes	Supine	Hip extension, neutral knee	2-examiners Bledsoe knee brace Anatomical landmark Standard GM	Passive	Compensation movements	APT	2 testing sessions (1–2 days apart) No detail	No detail	ICC = 0.97
			Supine	Hip extension, 90° knee flexion				No detail			ICC = 0.97
			Supine	Hip flexion, neutral knee				PPT			ICC = 0.90
			Supine	Ankle dorsiflexion, neutral knee			Maximun ROM	No detail			ICC = 0.98
			Prone	Ankle dorsiflexion, 90° knee flexion				No detail			ICC = 0.93
Witvrouw et al. [12]	No detail	No detail	Supine	Hip flexion, knee flexion	2-examiners Anatomical landmark Standard GM	Passive	No detail	No detail	No detail	Based on the study by Gogia et al. [68]	No detail
			Supine	Hip abduction			Compensation movements	Hip rotation			
			Prone	Knee flexion			Maximum ROM	No detail			
			Standing	Ankle dorsiflexion, neutral knee			Compensation movements	Heel on the floor			
Steinberg et al. [69]	No detail	No	Prone	Hip extension, knee extension	2-examiners Anatomical landmark Standard GM	Active	Compensation movements	Stabilized pelvis	Two testing sessions (1 day apart) consecutive days No detail	No detail	Pearson r = 0.91
			Supine	Hip abduction, knee extension		Passive		Hip rotation			Pearson r = 0.96
			Prone	Hip internal rotation, 90° knee flexion				Stabilized pelvis			Pearson r = 0.89
			Prone	Hip external rotation, 90° knee flexion				Stabilized pelvis			Pearson r = 0.89
			Supine	Hip flexion, knee flexion				PPT			Pearson r = 0.95
			Supine	Knee flexion				No			Pearson r = 0.93
			Supine	Ankle dorsiflexion, neutral knee		Passive	Compensation movements	Ankle and foot neutral position			Pearson r = 0.90
			Supine	Ankle plantarflexion, neutral knee							Pearson r = 0.91
Bradley and Portas [50]	No detail	No detail	Prone	Hip extension, neutral knee	2-examiners Reflective skin markers Software for 2-dimensional image-based analysis Video camera	Passive	Feeling of stretching	No detail	No detail 1 rep	No detail	ICC Hip = 0.92
			Supine	Hip flexion, flexion knee							
			Supine	Knee flexion, hip flexion							ICC knee = 0.95
			Supine	Knee extension, hip flexion							
			Supine	Ankle plantarflexion, neutral knee							ICC ankle = 0.91
			Supine	Ankle dorsiflexion, neutral knee							
Pua et al. [70]	No detail	No detail	Supine	Hip extension, 80° flexion knee	1-examiner Strap Anatomical landmark Extendable GM Electronic inclinometer	Passive	Firm end sensation Presence of pain	PPT, flattened lumbar spine	2 testing sessions (at least 1 week) 2 reps	No detail	MDC (90% IC) = 10.5°
			Supine	Hip extension, knee unconstrained							MDC (90% IC) = 11°
			Supine	Hip abduction, neutral knee				Stabilized pelvis			MDC (90% IC) = 7.3°
			Sitting	Hip internal rotation				No detail			MDC (90% IC) = 7.8°
			Sitting	Hip external rotation							MDC (90% IC) = 7.1°
			Supine	Hip flexion, flexion knee				APT, contralateral hip flexion			MDC (90% IC) = 8.2°
Bozic et al. [54]	25 min/group muscle	Yes	Supine	Hip abduction, neutral knee	2-examiners (a) Anatomical landmark Kinanthropometry, ruler and protractor Trigonometric calculations (b) Reflective markers 3D kinematic analysis system	Passive	Maximum ROM	No detail	2 testing sessions (1 week apart) 3 reps	Concurrent validity 3D kinematic analysis system vs Field methods ICC: 0.66 to 0.96 CV: 0.8 to 3.5%	ICC = 0.87; CV = 3.4%
			Supine	Hip flexion, neutral knee							ICC = 0.87; CV = 2.1%
			Standing	Single-legged knee bend		Active					ICC = 0.57; CV = 3.9%
			Standing	Sideward leg splits		Active					ICC = 0.89; CV = 2.3%
			Sitting	Sit and reach		Passive					ICC = 0.94; CV = 6.7%
			Standing	Sideways leg splits		Active					ICC = 0.88; CV = 2.4%
			Standing	Lengthwise leg splits		Active					ICC = 0.85; CV = 3%
Nussbaumer et al. [56]	No detail	No detail	Supine	Hip adduction, neutral knee	2-examiners (a) Anatomical landmark Standard GM Longitudinal axis (b) Anatomical landmark and sensor location ETS (electromagnetic tracking system)	Passive	Force application	No detail	2 testing sessions (1 week apart) 3 reps	Concurrent validity LOA: 3.3° ICC:0.53	GM (ICC = 0.84; CV = 6.7%) ETS (ICC = 0.82; CV = 6.3%)
				Hip abduction, knee extension						Concurrent validity LOA: 1.9° ICC:0.93	GM (ICC = 0.92; CV = 5.8%) ETS (ICC = 0.94; CV = 5.6%)
				Hip internal rotation, 90° hip and knee flexion						Concurrent validity LOA: 8.1° ICC:0.87	GM (ICC = 0.95; CV = 7.7%) ETS (ICC = 0.90; CV = 10.2%)
				Hip external rotation, 90° flexion hip and knee						Concurrent validity LOA: 3.5° ICC: 0.54	GM (ICC = 0.91; CV = 5.2%) ETS (ICC = 0.93; CV = 5.1%)
				Hip flexion, flexion knee						Construct validity (not differ between FAI and control) Concurrent validity (LOA: 18.9°; ICC: 0.44)	GM (ICC = 0.91; CV = 3.1%) ETS (ICC = 0.94; CV = 2.6%)
Fourchet et al. [53]	No detail	No detail	Supine	Hip abduction, neutral knee	Two examiners Anatomical landmarks Hand-held dynamometer Force application Video capture digital Digital motion analysis software	Passive	Force application	No detail	2 testing sessions (3 days apart) No detail	No detail	CV (90% IC) = 7.2%; ICC = 0.85
				Hip extension, knee flexion							CV (90% IC) = 2.6%; ICC = 0.51
				Hip internal rotation							CV (90% IC) = 9.6%; ICC = 0.92
				Hip external rotation							CV (90% IC) = 12.4%; ICC = 0.91
				Knee flexion, neutral hip							CV (90% IC) = 8.3%; ICC = 0.86
				Knee extension, 90° hip flexion							CV (90% IC) = 2.6%; ICC = 0.51
			Prone	Ankle dorsiflexion, neutral knee							CV (90% IC) = 4.5%; ICC = 0.93
				Ankle dorsiflexion, 90° knee flexion							CV (90% IC) = 5.7%; ICC = 0.66
Tainaka et al. [71]	No detail	No detail	Prone	Hip extension, neutral knee	1-examiner Anatomical landmarks Standard GM	Active	No detail	Stabilized pelvis and spine	2 testing sessions (1-week apart) 3 reps	No detail	Pearson r > 0.85
			Supine	Hip adduction							
				Hip abduction, neutral knee							
				Hip internal rotation, 90° hip and knee flexion							
				Hip external rotation, 90° flexion hip and knee							
				Hip flexion, knee flexion							
Cejudo et al. [45]	1 min	Yes	Supine	Hip extension, knee flexion	Two examiners Lumbar support “Lumbosant” (Imucot Traumatología SL, Murcia, Spain) Longitudinal axis (imaginary bisector line) Inclinometer with atelescopic rod	Passive	Firm end sensation Compensatory movements (lumbar spine, pelvis or lower limb) Feeling of stretching	APT	3 testing sessions (2-week apart) 2 or 3 (variation > 5%)	Content validity by American medical organizations	SEM = 1.3°; MDC = 3.7°; ICC = 0.96 Cejudo et al. [63]
			Supine	Hip adduction, 90° knee flexion				Transversal pelvis rotation			SEM = 1.8°; MDC = 4.5°; ICC = 0.92 Unpublished data
			Supine	Hip abduction, neutral knee				Frontal pelvis rotation, contralateral knee extension, transversal hip rotation			SEM = 1.8° MDC = 5.5°; ICC = 0.93; Cejudo et al. [63,72]
			Supine	Hip abduction, 90° hip and knee flexion				Transversal pelvis rotation			SEM = 2.1°; MDC = 5.8°; ICC = 0.99 Cejudo et al. [72]
			Supine	Hip flexion, extension knee				PPT, knee flexion, hip rotation, contralateral hip flexion			SEM = 1.9°; MDC = 6.1°; ICC: 0.91; Cejudo et al. [63]
			Supine	Hip flexion, flexion knee							SEM = 2.5°; MDC = 6.2°; ICC = 0.90 Cejudo et al. [63]
			Supine	Knee flexion, neutral hip				APT, hip rotation			SEM = 2.8°; MDC = 6.9°; ICC = 0.89; Cejudo et al. [63]
			Prone	Hip internal rotation, neutral hip and 90° knee flexion				Transversal pelvis rotation, hip abduction			SEM = 2.5°; MDC = 6.8°; ICC = 0.92 Unpublished data
			Prone	Hip external rotation, neutral hip and 90° knee flexion				Transversal pelvis rotation, hip abduction			SEM = 2.5°; MDC = 6.8° ICC = 0.92 Unpublished data
			Standing	Ankle dorsiflexion, neutral knee				Heel on the floor, Knee flexion			SEM = 1.7°; MDC = 4.7°; ICC = 0.95 Cejudo et al. [63]
			Standing	Ankle dorsiflexion, Knee flexion				Heel on the floor			SEM = 1.8°; MDC = 5°; ICC = 0.95 Cejudo et al. [63]
Shah et al. [11]	No detail	Yes	Prone	Hip extension	2-examiners Lateral midline of the thigh and horizontal axis of the body 2-examiners Bony landmark Standard GM	Passive	No detail	No details	2 testing sessions 1 rep	No detail	ICC = 0.62
			Supine	Hip flexion			Compensation movements Maximum ROM				ICC = 0.77
				Hip internal rotation, 90° flexion hip and knee				Transversal pelvis rotation, lumbar lateral flexion			ICC = 0.77
				Hip external rotation, 90° flexion hip and knee							ICC = 0.90
Grazette et al. [73]	No detail	No detail	Standing	Ankle dorsiflexion, knee flexion	Centimeters measurement units	Passive	Maximum ROM	Heel on the floor, knee flexion, foot pronation, foot supination, pelvic rotation, knee valgus or varus	2 testing sessions (3–7 days apart) No detail	No detail	ICC = 0.95 CV = 35.9°
			Supine	Medial hip rotation, 90˚ hip and knee	2-examiners Standard GM	Passive	Firm end sensation	No detail			ICC = 0.72 CV = 8.3°
			Prone	Hip internal rotation		Passive					ICC = 0.70 CV = 36.5°
			Supine	Hip external rotation		Active					ICC = 0.82 CV = 49°
			Supine	Knee extension (Hamstring 90/90)		Passive					ICC = 0.47 CV = 65.3°

GM, goniometer; ORR, orthoranger; APT, anterior pelvis tilt, PPT, posterior pelvis tilt; CV, coefficient of variation at 95% confidence intervals; ICC, intraclass correlation coefficient; SEM, standard error of the mean; MDC, minimal detectable change at 95% confidence intervals (CI).
